# Therapeutic potency of bee pollen against biochemical autistic features induced through acute and sub-acute neurotoxicity of orally administered propionic acid

**DOI:** 10.1186/s12906-016-1099-8

**Published:** 2016-04-23

**Authors:** Huda S. Al-Salem, Ramesa Shafi Bhat, Laila Al-Ayadhi, Afaf El-Ansary

**Affiliations:** Biochemistry Department, Science College, King Saud University, P.O. Box 22452, 11495 Riyadh, Saudi Arabia; Department of Pharmaceutical Chemistry, College of Pharmacy, King Saud University, Riyadh, Saudi Arabia; Autism Research and Treatment Center, Riyadh, Saudi Arabia; Shaik AL-Amodi Autism Research Chair, King Saud University, Riyadh, Saudi Arabia; Department of Physiology, Faculty of Medicine, King Saud University, Riyadh, Saudi Arabia; Medicinal Chemistry Department, National Research Centre, Dokki, Cairo Egypt

**Keywords:** Autism, Propionic acid, Neurotransmitters, Interferon gamma, Caspase 3, Bee pollen

## Abstract

**Background:**

It is now well documented that postnatal exposure to certain chemicals has been reported to increase the risk of autism spectrum disorder. Propionic acid (PA), as a metabolic product of gut microbiotaandas a commonly used food additive, has been reported to mediate the effects of autism. Results from animal studies may help to identify environmental neurotoxic agents and drugs that can ameliorate neurotoxicity and may thereby aid in the treatment of autism. The present study investigated the ameliorative effects of natural bee pollen against acute and sub-acute brain intoxication induced by (PA) in rats.

**Methods:**

Twenty-four young male Western Albino ratswere enrolled in the present study. They were classified into four equal groups, eachwith6 rats. The control group received only phosphate buffered saline; the oral buffered PA-treated groups (II and III) received a neurotoxic dose of 750 mg/kg body weight divided in 3 dose of 250 mg/kg body weight/day serving asthe acute group and 750 mg/kg body weight divided in 10 equal dose of 75 mg/kg body weight/day as the sub-acute group. The fourth group received 50 mg bee pollen for 30 days after PA-acute intoxication.

**Results:**

The obtained data showed that the PA-treated groups demonstrated multiple signs of brain toxicity, as indicated by a depletion of serotonin (5HT), dopamine and nor-adrenaline, together withan increase in IFN-γ and caspase 3. Bee pollen was effective in ameliorating the neurotoxic effect of PA. All measured parameters demonstrated minimal alteration in comparison with thecontrol animal than did those of acute and sub-acute PA-treated animals.

**Conclusions:**

In conclusion, bee pollen demonstrates anti-inflammatory and anti-apoptotic effects while ameliorating the impaired neurochemistry of PA-intoxicated rats.

## Background

A developmental state is the most dominant, host-related factor that affects its response to environmental toxicants. The immature rat brain is morevulnerable toneurotoxic agents than is the adult animal. The single most critical factor of the pattern of damage induced by neurotoxic agents is thetiming of exposure. As later stages of neurodevelopment depend upon the successful completion of early stages, minor disturbances during brain development may cause drastic damage in the future, and neurodevelopmental process are differentially sensitive to specific neurotoxins [1]. Additionally, because different brain regions develop at differenttimes during prenatal and postnatal life, a chemical may produce impairment in different functionaldomains, depending upon the time of exposure [1].

Recently, the behavioral, neuropathological and biochemical abnormalities following exposure to propionic acid (PA) neurodevelopment toxicity were recorded as etiological factors of autism. As this short chain fatty acid is used as a food additive and is produced by certain bacterial species that are known as propionibacteria(e.g., *Clostridium difficile* and *Klebsiella pneumonia*), it may provide a link between dietary, enterobacterial metabolites, and a genetic predisposition for the subsequent etiology of persistent autistic features in exposed rat pups [2, 3].

A growing body of evidence implicateshyperserotonemia, immunological disturbances, oxidative stress and poor detoxification ability together with elevation of pro-apoptotic markers in the peripheral blood of autistic patients [3–8]. Different animal models have been produced to investigate the environmental contribution, possible causes, and potential treatments of autism. Among the several animal models that have so far been tested, the rat model appears to be an excellent translational system because detailed data are already available on the genetics and behavioral phenotyping of various strains [9]. The etiology ofpersistent autistic features in rat pups were recorded through a panel of biomarkers related to oxidative stress [2, 9], neuroinflammation [3], and abnormal neurotransmission [10], together with autistic behavioral changes [11].

Bee pollen is a natural product that is composed of amino acids, lipids, flavinoids, vitamins and micronutrients. It demonstrates antifungal, antimicrobial, anti-inflammatory, and immunostimulating effects [12, 13]. Pollen is a rich source of fat-soluble vitamins, such as vitamin A, E and D, together with water-soluble vitamins, such as B1, B2, B6, and C. Bee pollen is known to have detoxification activity and can remove heavy metals (e.g., mercury and lead) and drugs (e.g., antibiotics and anti-inflammatory preparations). Pollen also demonstrates anti-inflammatory mechanisms through the inhibition of the activities of cyclooxygenase and lipoxygenase, the enzymes that are responsible for the conversion of arachidonic acid intotoxic compounds as prostaglandin and leukotrienesas inducers of acute and chronic inflammatory conditions in different tissues [14, 15].

The recently recorded apitherapeutic mechanism of pollen is attributed to its antimicrobial activity and potency to induce regeneration of damaged tissues [16]. It has also been shown that the ethyl alcohol extract of pollen has antibiotic activity against Gram-positive pathogenic bacteria,including *Klebsiella pneumonia (A propionobacteria)*and *Pseudomonas aeurgionsa,* and against fungi, such as *Candida albicans*. Theresponsibility for this activity lies in flavonoids and phenolicacids [17, 18]. The antioxidant effects of these components are largely related to their free radical scavenging activity. It is the most important components that can treat oxidative stress as an etiological factor and a potential treatment target of autism [19].

This information initiates our interest to test the therapeutic effects of bee pollen on selected biomarkers that are known to be clinically impaired in autistic patients and in a rodent model of autism.

## Methods

The experimental assays for this study were performed on 24 young (approximately 21 days old) male western albino rats (45 to 60 g). The animals were fed on standard pellet diet, water ad libitum and were maintained in a controlled environment under standard conditions of temperature and humidity with an alternating light- and dark-cycle. Rats were obtained from the animal house of the pharmacy college, King Saud University, and were randomly assigned to four groups of six rats each. The first group consisted of rats to which only phosphate buffered saline was administered and were used as a control group. The second group of rats were given an oral neurotoxic dose (750 mg PA/kg body weight over 3 days at a dose of 250 mg PA/kg body weight/day) and served as the acutely treated group. The third group was treated with 750 mg PA/kg body weightover 10 days at a dose of 75 mg PA/kg body weight/day) and served as the sub-acutely treated group. The fourth group received bee pollen (50 mg/kg body weight/day for 30 days) [12]. Bee pollen used in the present study is first elite product, 100 % natural, imported for Wadi Al-Nahilone of the largest marketing company in Saudi Arabia (www.wadialnahil.net). The four groups of rats were housed under controlled temperature (21 ± 1 °C) with ad libitum access to food and water. The protocol of the present work was approved by the Ethics Committee at the King Saud University, and all experiments were performed in accordance with the guidelines of the National Animal Care and Use Committee.

### Tissue preparation

At the end of the experiment, the rats were anesthetizedwith carbon dioxide and decapitated. The brains were removed from the skull and were dissected into smallpiecesto be homogenized either in distilled water (10 times w/v) (For the assay of IFγ and caspase-3) or perchloricacidforthe neurotransmitter assay.

#### Assay of neurotransmitters (NA, DA and 5HT)

The concentrations of NA, DA, 5-HT were determined in brain homogenates using high-performance liquid chromatography with electrochemical detection (HPLC-ED) [20]. Brain tissue was homogenized in 150 μl 0.1 M perchloric acid containing 0.4 mM sodium metabisulphiteusingan ultrasonic cell disrupter. Thehomogenates were then centrifuged at 10,000 x g at 4 °C for 25 min, and the supernatants were filtered through a 0.22 m filter (Sigma) and frozen at −70 °C until analysis.

Filtrate was injected into the HPLC system, which consisted of a quaternary gradient delivery pump Model HP 1050 (Hewlett-Packard), a sample injector Model 7125 (Rheodyne, Berkeley), and an analytical column ODS 2 C18, 4.6 × 250 mm (Hewlett-Packard) that was protected by guard column (Lichnospher 100 RP-18, 4 × 4 mm) with a particle size of 5 μm (Hewlett-Packard). The mobile phase was comprised of 0.15 M sodium dihydrogen phosphate, 0.1 mM EDTA, 0.5 mM sodium octanesulphonic acid, 10–12%methanol (v/v) and 5 mM lithium chloride. The mobile phase was adjusted to pH 3.4 with phosphoric acid, filtered through 0.22 m filter (Sigma) and degassed with helium. A column temperature of 32 °C and a flow rate of 1.4 ml/min were used.

The electrochemical detector model HP 1049 A (Hewlett-Packard) with a glassy carbon workingelectrode was used at a voltage setting of +0.65 Vfor monoamines. The detector response was plotted and measured using a chromate-integrator. The concentration of NA, DA, 5-HT in each sample was calculated from the integrated chromatographic peak area and expressed asng/100 mg wet tissue.

#### Assay of interferon gamma

IFNγ was measured using an ELISA kit, a product of Thermo Scientific (Rockford, IL, USA), according to the manufacturer’s instructions. A polyclonal antibody specific for human IFNγwas pre-coated onto a 96-well microplate. IFNγ in standards and samples were sandwiched by the immobilized antibody and biotinylated polyclonal antibody specific for IFNγ, which was then recognized by a streptavidin-peroxidase conjugate. All unbound material was then washed away, and a peroxidase enzyme substrate was added. The color development is stopped, and the intensity of the color is measured at 550 nm and subtracted from absorbance at 450 nm. The minimum level of IFNγ detected by this product is less than 2 pg/ml.

### Assay of Caspase3

Caspase3 was measured using an ELISA kit, a product of Cusabio (Cusabio, Wuhan, China). The microtiter plate provided in this kit was pre-coated with an antibody specific for caspase3. Standards or samples were then added to the appropriate microtiter plate wells with a biotin-conjugated antibody preparation specific for caspase3. After that, avidin conjugated to horseradish peroxidase (HRP) was added to each microplate well and incubated. A TMB (3, 3’, 5, 5’tetramethyl-benzidine) substrate solution was then added to each well. Only the wells that contained caspase3, biotin-conjugated antibody, and enzyme-conjugated avidin would exhibit a change in color. The enzyme-substrate reaction was terminated by the addition of a sulfuric acid solution, and color change was measured spectrophotometrically at a wavelength of 450 nm ± 2 nm. The concentration of caspase 3 in the samples was then determined by comparing the optical density (O.D.) of the samples withthe standard curve.

### Statistical analysis

The data were analyzed using the statistical package for the social sciences (SPSS, Chicago, IL, USA). The results were expressed as the mean ± S.D. All statistical comparisons between the control and PA and pollen-treated rat groups were performed using a one-way analysis of variance (ANOVA) test complemented with the Dunnett test for multiple comparisons. Significance was assigned at the level of *P* <0.05. A receiver operating characteristics curve (ROC) analysis was performed. The area under the curve (AUC), cutoff values, and degree of specificity and sensitivity were calculated. The area under the curve (AUC) provides a useful metric for comparing different biomarkers, whereas an AUC value close to 1 indicates an excellent predictive marker, a curve that lies close to the diagonal (AUC = 0.5) has no diagnostic utility. An AUC close to 1 is always accompanied by satisfactory values of specificity and sensitivity of the biomarker. Pearson’s correlations were performed between the measured parameters.

## Results

**Fig. 1:**
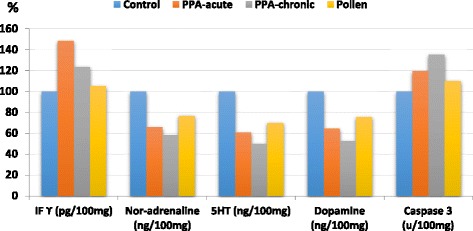
Percentage change of all parameters in all groups in comparisonwith the control

The brain homogenates levelsof IFγ, nor-adrenaline, 5HT, dopamine and caspase-3 in addition to their percentage change relative to control of all the tested groups,are presented in Table 1. As revealed in Fig. 1, the acute PA–treated group exhibited a significant increase of IFγ (148.6 %) and caspase-3 (119.7 %), with a concomitant decrease of the three measured neurotransmitters, NA (66.1 %), DA (64.8 %) and 5HT (61.00 %), in comparison with thecontrol. Likewise, the treatment with a subacute dose of PA in group III significantly increased IFγ (123.9 %) and caspase-3 (119.7 %),with a drastic decrease in NA (58.5 %), DA (52.9 %) and 5HT (50.1 %)in comparison withthe control group. Comparison between group II and group III showed thatsuba-cute mode of treatment provedto be more neurotoxin than acute one. (Table 1). The brain homogenate of bee pollen treated animals also showed improvement in all the tested parameters as shown in Table 1.

**Table 1:** The mean ± S.D and percentage changes of the four measured parameters in brain homogenates of PPA-acute and sub-acuteintoxicated rats and pollen-treated rats in comparisonwiththecontrol animals

Parameter	Group	N	Mean ± S.D.	Percent change	*P* value*	*P* value**
IF ϒ (pg/100 mg)	Control	6	82.82 ± 7.52	100.00		
	PPA-acute	6	123.05 ± 6.58	148.57	0.001	
	PPA-sub-acute	6	102.60 ± 5.08	123.87	0.001	0.001
	Pollen	6	87.40 ± 2.09	105.52	0.203	0.001
Nor-adrenaline (ng/100 mg)	Control	6	4.92 ± 0.61	100.00		
	PPA-acute	6	3.25 ± 0.36	66.12	0.001	
	PPA-sub-acute	6	2.88 ± 0.32	58.49	0.001	0.086
	Pollen	6	3.79 ± 0.15	77.18	0.005	0.013
5-HT (ng/100 mg)	Control	6	6.20 ± 0.78	100.00		
	PPA-acute	6	3.78 ± 0.56	61.00	0.001	
	PPA-sub-acute	6	3.11 ± 0.18	50.07	0.001	0.029
	Pollen	6	4.36 ± 0.42	70.36	0.005	0.069
Dopamine (ng/100 mg)	Control	6	21.12 ± 2.72	100.00		
	PPA-acute	6	13.69 ± 0.80	64.81	0.001	
	PPA-sub-acute	6	11.17 ± 1.29	52.92	0.001	0.002
	Pollen	6	16.09 ± 0.71	76.20	0.005	0.001
Caspase 3 (u/100 mg)	Control	6	112.66 ± 4.20	100.00		
	PPA-acute	6	134.79 ± 3.27	119.65	0.001	
	PPA-sub-acute	6	152.57 ± 8.97	135.43	0.001	0.003
	Pollen	6	124.23 ± 2.89	110.28	0.001	0.001

*P value between control group and other groups

**P value between PPA-acute group and other groups

**Fig. 2:**
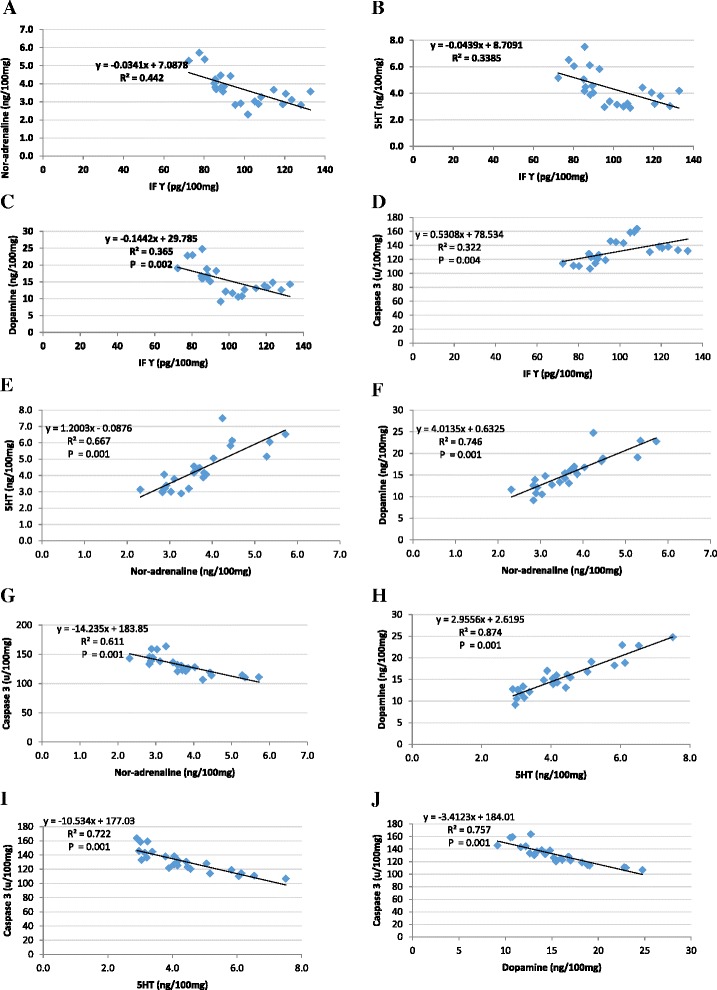
Pearson’s positive and negative correlations with best-fit line curves between the measured parameters. **a** IFϒ and nor-adrenaline (−ve), **b** IFϒ and 5-HT (−ve), **c** IFϒ and dopamine(−ve), **d** IFϒ and caspase 3 (+ve), **e** nor-adrenaline and 5-HT(+ve), **f** noradrenaline and dopamine (+ve), **g** nor-adrenaline and caspase 3(−ve), **h** 5-HT and dopamine (+ve), **i** 5-HT and caspase 3 (− ve), and **j** dopamine and caspase 3 (−ve)

Regarding the Pearson's correlations between the measured parameters, Table 2 and Fig. 2 demonstrate the significantpositive correlations betweenIFϒ and caspase 3 (*R* = 0.567; *p* = 0.004), nor-adrenaline and 5HT (*R* = 0.817; *p* = 0.001), nor-adrenaline and dopamine (*R* = 0.864; *p* = 0.0015) and 5HT and dopamine (*R* = 0.935; *p* = 0.001). IFϒ was significantly associated with nor-adrenaline (*R* = −0.665; *p* = 0.001), 5HT (*R* = −0.582; *p* = 0.003) and dopamine (*R* = −0.604; *p* = 0.002). There was also a significant negative correlation between 5HT ~ caspase 3 (*R* = −0.870; *P* = 0.001) and dopamine ~ caspase 3 (*R* = −0.870; *P* = 0.001).

**Table 2:** Pearson correlations between the measured parameters

Parameters	R (Person correlation)	Sig.	
IFϒ ~ Nor-adrenaline	−0.665^c^	0.001	N^b^
IFϒ ~ 5-HT	−0.582^c^	0.003	N^b^
IFϒ ~ Dopamine	−0.604^c^	0.002	N^b^
IFϒ ~ Caspase 3	0.567^c^	0.004	P^a^
Nor-adrenaline ~ 5-HT	0.817^c^	0.001	P^a^
Nor-adrenaline ~ Dopamine	0.864^c^	0.001	P^a^
Nor-adrenaline ~ Caspase 3	−0.781^c^	0.001	N^b^
5-HT ~ Dopamine	0.935^c^	0.001	P^a^
5-HT ~ Caspase 3	−0.850^c^	0.001	N^b^
Dopamine ~ Caspase 3	−0.870^c^	0.001	N^b^

^a^Positive Correlation

^b^Negative Correlation

^c^Correlation is significant at the 0.01 level

**Fig. 3:**
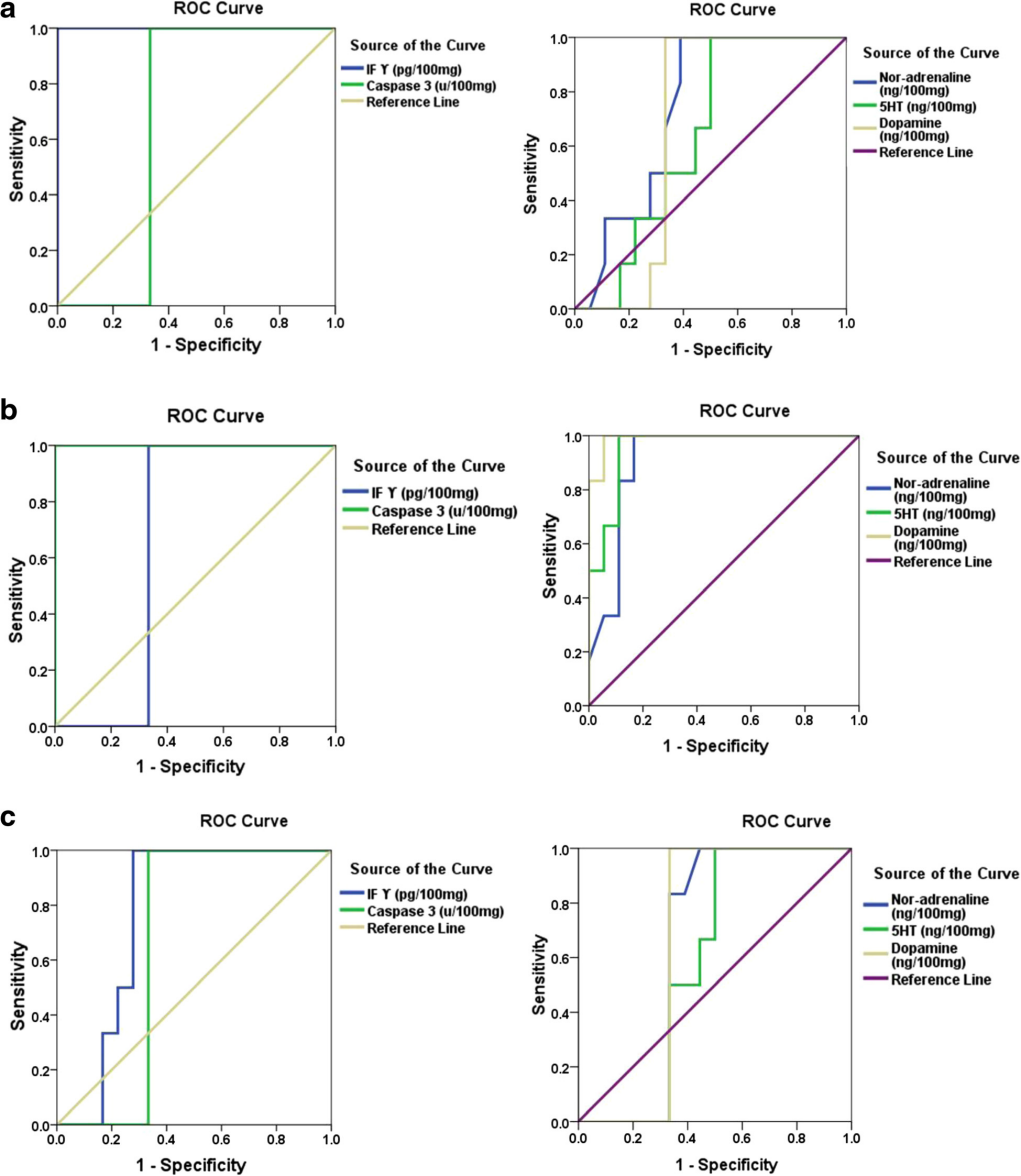
ROC Curve of **a** all parameters in the PPA-acute group, **b** all parameters in the PPA-sub-acute group, and **c** all parameters in the pollen group

Receiver operating characteristics curves are collectively presented as curve A, B and C in Fig. 3. Area under the curve (AUC), cutoff values, sensitivity and specificity are listed in Table 3.

**Table 3:** ROC curve of the measured parameters in acute, sub-acute and pollen-treated groups in all groups

	Group	Area under the curve	Best Cutoff value	Sensitivity %	Specificity %
IFϒ (pg/100 mg)	PPA-acute	1.000	111.370	100.0 %	100.0 %
	PPA- sub-acute	0.667	94.260	100.0 %	66.7 %
	Pollen	0.769	91.450	100.0 %	72.2 %
Nor-adrenaline (ng/100 mg)	PPA-acute	0.741	3.685	100.0 %	61.1 %
	PPA- sub-acute	0.912	3.360	100.0 %	83.3 %
	Pollen	0.653	3.510	100.0 %	55.6 %
5-HT (ng/100 mg)	PPA-acute	0.639	4.450	100.0 %	50.0 %
	PPA- sub-acute	0.954	3.585	100.0 %	88.9 %
	Pollen	0.593	3.840	100.0 %	50.0 %
Dopamine (ng/100 mg)	PPA-acute	0.676	15.005	100.0 %	66.7 %
	PPA- sub-acute	0.991	12.920	100.0 %	94.4 %
	Pollen	0.667	15.005	100.0 %	66.7 %
Caspase 3 (u/100 mg)	PPA-acute	0.667	129.310	100.0 %	66.7 %
	PPA- sub-acute	1.000	140.835	100.0 %	100.0 %
	Pollen	0.667	129.310	100.0 %	66.7 %

## Discussion

Different animals can be used for acute and sub-acute testing of toxicity, but they may vary with respect to the route of toxin administration. For oral administration, the preferred rodent species is the albino rat (Wistar), and the test substance is usually given in a single dose by gavage [21].

Table 1 demonstrates the acute and sub-acute toxic effects of PA together with the therapeutic potency of bee pollen. Elevated IFN- γ can easily show the neurotoxic effect of PA [22]. It was shown that IFN-γ–activated astrocytes become neurotoxic through the activation of STAT 3 signal transducer, and so STAT3 inhibitors may have an anti-neurotoxic effect. Based upon this mechanism, the therapeutic effect of bee pollen, presented in Table 1, demonstrated a non-significant difference between pollen-treated group and control animals (*P* <0.203),which can be explained by its specific inhibitory mechanism ofangiogenic processes, among which is STAT3 inhibition. Moreover, it can be related to the anti-inflammatory effect of bee pollen. Flavonoids, as major components of pollen, are efficient in decreasing the expression of the inflammatory signaling pathway. This always helps to inhibit the excessive release of nitric oxide and COX-2 expression through the prevention of NF-kBactivation [23–25]. This can be supported by the fact that NO, COX-2 and NF-kB activation are all recorded as etiological mechanisms related to autism [26–28]. Elevated IFN-γ can be related to the recorded 5HT depletion as a neurotransmitter known to be depleted in a PA-induced rodent model of autism [3]. IFN-γ induces indoleamine 2,3-dioxygenase as an enzyme that catalyzes the breakdown of tryptophan, resulting in serotonin depletion [29].

It was generally believed that while brain function is associated with hunger or satiety changes, its functionis independent of metabolic changes associated with food consumption. However, in 1971, Fernstrom and Wurtman [30] proved that under certain conditions, the protein-to-carbohydrate ratio of a meal could affect the concentration of a particularbrain neurotransmitter. For example, the brain turnover of two catecholamine neurotransmitters, dopamine and norepinephrine, can be greatly affected by ingestion of their amino acid precursor, tyrosine, when neurons that release these monoamines are firing frequently. In addition, serotonin, a neurotransmitter involved in the regulation of a variety of brain functions, such as sleeping, pain sensitivity, aggression, and patterns of nutrient selection, have also been shown to be affected by dietary constituents, which are given either as ordinary foods or in purified supplement. Based upon this,it can be suggested that neurotransmitters could be affected by precursor availability or other peripheral factors that are governed by food consumption [31].

Table 1 demonstrates theneurotoxic effect of PA altering the level of DA, 5HT, and NA. Moreover, the therapeutic effect of bee pollen was clear as it ameliorated the recorded neurotoxic effect of PA, thereby demonstrating less significant differencesin comparisonwiththe control. This effect can be attributed to the nutritional fact that pollen contains 22.7 % of protein on average, including 10.4 % of essential amino acids,among which is phenylalanine (precursor of tyrosine) and tryptophan. Bee pollen contains 0.69 ± 0.003 % and 2.693 ± 0.476 %of free and total tryptophan, respectively [32]. The reported ameliorating effect of pollen can be attributed to its tryptophan content, as clinical evidence indicates that social impairment, as a core symptom in autism, is related to inadequate brain 5-HT stores [33, 34] and thattryptophandepletionworsens autism symptoms. This can also be supported by reports from parents that their autistic children consume less tryptophan than do their peers. A recent study by Zhang et al. [35] demonstrates that mouse sociability is reduced by acute tryptophan depletion and can be enhanced by tryptophan supplementation.

**Fig. 4:**
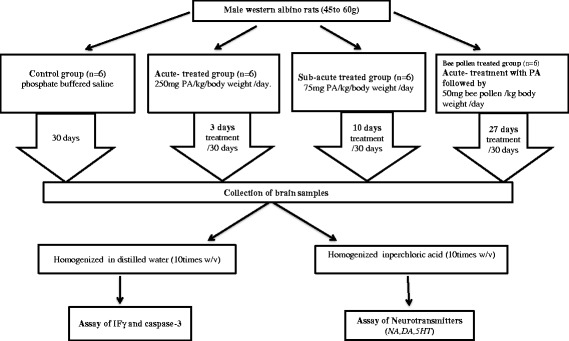
Schematic presentation of the experimental work demonstrating treatment of different studied groups

Apoptosis, as a type of programmed cell death, requires specialized cellular machinery, including a family of cysteine proteases known as caspases [36]. Among these caspases, caspase-3 is a potent effector of neuronal death during brain development and under certain pathological conditions [37]. Based upon this fact, the neurotoxic effect of PA together with the therapeutic effect of bee pollen can easily be observed in Table 1 and Fig. 4. While acute and sub-acute PA neurotoxicity demonstrated an almost 20 and 35.43 % increase in the concentration of caspase3, respectively, bee pollen demonstrated that a reduction in caspase 3 was only 10 % higher in comparisonwiththecontrol. Increase of caspase 3 as a marker of PA neurotoxicity can be related to the elevation of amyloid beta Aβ that was previously reported in the autistic brain. Elevation of amyloid precursor protein (APP) is usually followed by caspase-3 activation, which causes both apoptosis and the proteolytic processing of APP that results in Aβformation [38, 39]. The effect of bee pollen on caspase 3 can be attributed toitsantioxidant effect of flavonoid. It is well documented that flavonoids might protect against apoptosis of hippocampal neurons through suppressing caspasesandthemitochondrial pathway [40].

Based upon the results of the present study, bee pollen can be suggested as a treatment strategy for autistic children that suffer from detoxification deficiencies, demonstrated chronic inflammation and abnormal gut microbiota [41–44]. This suggestion is supported with some recent studies that demonstrated the detoxifying [45], anti-inflammatory [46, 47], and anti-microbial effects of bee pollen [13, 17, 18].

Table 2 and Fig. 1 demonstrate the positive correlations between the three measured neurotransmitters (5-HT, dopamine and nor-adrenaline). This can ascertain the impairment of brain neurochemistry by PA and also the therapeutic effects of bee pollen. A positive correlation between IFN-γ and caspase 3 demonstrates the role of neuroinflammation in neuronal loss. This can find support in the work of Lopez-Ramirez et al. [48], which proves that high TNF-α and IFN-γ levels were associated with caspase-3/7 activation, which is directly related to blood-brain barrier damage through the alteration of the phenotype and function of brain endothelial cells. Conversely, negative correlations between the impaired neurotransmitters and bothIFN-γ and caspase 3 suggest that amelioration of neurotransmitters through the availability of their amino acids precursors (e.g., tyrosine and tryptophan) in bee pollen can help to reduce the elevated concentrations of IFN-γ and caspase-3 as markers of neuroinflammation and apoptosis, respectively.

Table 3 and Fig. 2 demonstrate the area under the curve (AUC), specificity and sensitivity of the measured parameters in acute and sub-acute PA intoxicated rats together with bee pollen–treated animals. Asall measured parameters recorded satisfactory values of AUC, sensitivity and specificityserve as markers for sub-acute PA neurotoxicity and as less predictive values for the ameliorating effects of bee pollen (AUC range of 0.6–0.769), while IFϒ is suggested to be a good marker for acute PA toxicity given that the AUC is equal to 1 and that it is 100 % specific and sensitive [49].

## Conclusion

Acute and sub-acute orally administered propionic acid resulted in changes in biochemical parameters. The alteration of neurotransmitters, cytokines and pro-apoptotic markers observed can be related to oxidative stress induced by PA. Bee pollen, due to the biological properties of its components (in particular, phenolic compounds and amino acid composition), has been determined to exhibit strong free radical scavenging and antioxidant activity. Therefore, it has been concluded that bee pollen can be used safely to ameliorate oxidative stress, neuroinflammation, poor detoxification, and abnormal gut microbiota as mechanisms involved in the etiology of autistic features.

### Ethics approval and consent to participate

Present work was approved by the Ethics Committee at the King Saud University, and all experiments were performed in accordance with the guidelines of the National Animal Care and Use Committee.

### Consent for publication

Not applicable.

### Availability of data and materials

The datasets supporting the conclusions of this article are presented in this main paper. Animals were obtained from the animal house of the pharmacy college, King Saud University.

## Abbreviations

5HT: serotonin
ANOVA: one-way analysis of variance
APP: amyloid precursor protein
AUC: area under the curve
Aβ: amyloid beta
DA: dopamine
EDTA: ethylenediaminetetraacetic acid
HPLC-ED: high-performance liquid chromatography with electrochemical detection
HRP: horseradish peroxidase
IFN-γ: interferon gamma
NA: nor-adrenaline
O.D: optical density
PA: propionic acid
ROC: receiver operating characteristics curve
S.D: standard deviation
TNF-α: tumor necrosis factor alpha
